# Prevalence of heart failure and trends in its pharmacological treatment between 2000 and 2017 among very old people

**DOI:** 10.1186/s12877-024-05307-4

**Published:** 2024-08-24

**Authors:** Sofia Svahn, Leona Appelblad, Hugo Lövheim, Yngve Gustafson, Birgitta Olofsson, Maria Gustafsson

**Affiliations:** 1 Department of Medical and Translational Biology, Umeå University, Umeå, 901 87 Sweden; 2 Department of Community Medicine and Rehabilitation, Geriatric Medicine, Umeå University, Umeå, 901 87 Sweden; 3 Department of Nursing, Umeå University, Umeå, 901 87 Sweden; 4 Department of Diagnostics and Intervention, Orthopedics, Umeå University, Umeå, 901 87 Sweden

**Keywords:** Heart failure, Very old people, Cardiovascular drugs, Drug use

## Abstract

**Purpose:**

The aim of this study was to describe a population of very old people with heart failure (HF), to analyse the use of cardiovascular drugs over time, and to explore factors influencing cardiovascular drug treatment for this group.

**Methods:**

All participants with information regarding HF diagnosis were selected from the Umeå 85+/Gerontological Regional Database (GERDA). The people in GERDA are all ≥85 years old. Trained investigators performed structured interviews and assessments. Information regarding medications and diagnoses was obtained from the participants and from medical records. Medical diagnoses were reviewed and confirmed by an experienced geriatrician.

**Results:**

In this very old population, the prevalence of HF was 29.6% among women and 30.7% among men. Between 2000 and 2017, there was an increase in the use of renin-angiotensin (RAS) inhibitors (odds ratio [OR] 1.107, 95% confidence interval [CI] 1.072–1.144) and beta-blockers (BBs) (OR 1.123, 95% CI 1.086–1.161) among persons with HF, whereas the prevalence of loop diuretics (OR 0.899, 95% CI 0.868–0.931) and digitalis (OR 0.864, 95% CI 0.828–0.901) decreased (*p* < 0.001 for all drug classes). Higher age was associated with lower use of RAS inhibitors and BBs.

**Conclusion:**

In this HF population, the use of evidence-based medications for HF increased over time. This may be a sign of better awareness among prescribers regarding the under-prescribing of guidelines-recommended treatment to old people. Higher age associated with a lower prevalence of RAS inhibitors and BBs. This might indicate that further improvement is possible but could also represent a more cautious prescribing among frail very old individuals.

**Supplementary Information:**

The online version contains supplementary material available at 10.1186/s12877-024-05307-4.

## Introduction

Heart failure (HF) has been reported to affect about 1–2% of the population [1]. The condition is associated with high morbidity and mortality. Its prevalence increases with age, and it is a leading cause of hospitalisation among old people [2–5]. Old people have been poorly represented in randomised controlled trials (RCTs) for the management of HF, and even though there are some uncertainties regarding the effect of these treatments in the older population, the guidelines still apply to old people [6]. However, several studies have found that very old people are less likely to receive evidence-based treatment for HF [7–13].

Since their first publication, the European Society of Cardiology (ESC) guidelines have always recommended the use of renin-angiotensin (RAS) inhibitors and beta-blockers (BBs) for all patients with HF with reduced ejection fraction (HFrEF) and New York Heart Association (NYHA) class II–IV disease, as these drugs are known to reduce mortality and morbidity [1, 14–19]. Mineralocorticoid receptor antagonists (MRAs) have been recommended for NYHA class III–IV since the 1997 version of the guidelines [14], and for NYHA class II since the 2012 version [18]. The evidence for the same outcomes is weaker for loop diuretics. Loop diuretics have always been recommended to treat congestive symptoms, with the advice that the drug should be reduced to the lowest effective dose and, if possible, discontinued. Digitalis has also had a place in all HF guidelines as an additional option in patients with remaining symptoms [1, 14–19]. In addition to reducing mortality and hospital admission, adequate HF treatment may lead to relief of symptoms and improved quality of life [20].

Most previous studies describing very old people with HF and their drug treatment have been based on HF registries, while less has been published regarding real-life populations [13, 21, 22]. Due to the reported problems in implementing evidence-based treatment among old people with HF [23, 24], and because the majority of articles describing lower use of guideline-recommended treatment among old people are cross-sectional studies with data from only one time point [7, 8, 10, 11], we were interested in investigating time trends in drug use in a very old real-life population to see if the use of evidence-based therapy has changed over time.

## Aim

The aim of this study was to describe a population of very old people with HF, to analyse the use of cardiovascular drugs over time, and to explore factors influencing cardiovascular drug treatment for this group.

## Method

### Setting and participants

The data in this study were derived from the Umeå 85+/Gerontological Regional Database (GERDA), which started with the objective of investigating the general health and living conditions of very old people in northern Sweden. The database was initiated in the year 2000, and data collection was conducted every five years. This study used cross-sectional data from the database collected at four different time points: 2000–2002 (T1), 2005–2007 (T2), 2010–2012 (T3), and 2015–2017 (T4). Potential participants were identified by using the population register from the Swedish Tax Agency.

Every second 85-year-old and all individuals aged 90 and ≥95 years living in one urban or five selected rural municipalities were invited to participate. An information letter was sent to those selected, and they were contacted via telephone one week later. Informed consent was obtained from all included participants. If the individual was not personally able to give consent, for example due to substantial cognitive impairment, a close person/relative was also consulted about participation. This approach made it possible to include people living at home as well as in nursing homes, and people with and without cognitive impairment. Participants could be included at multiple time points, and were then considered unique cases for each time point. The Umeå 85+/GERDA study is described in greater detail in previous publications [25–27]. For this study, all participants with information regarding HF diagnosis were selected. In total, 2186 of the 2814 invited individuals were included, giving a participation rate of 77.7%.

### Procedure

Trained investigators conducted structured interviews with the participants during home visits. Physical tests were performed in addition to filling out questionnaires and assessment scales. The investigator was a medical doctor, medical student, nurse, or physiotherapist. For individuals with cognitive decline or living in nursing homes, the interviews were also held with partners, relatives, or staff.

Data regarding diagnoses, medical conditions, current drug use, and drug prescriptions were collected from the respondents and from medical records. The information on current drug use reported by the respondents was crosschecked with medical records. Information regarding doses and use of *pro re nata* medication was not available. An experienced geriatrician reviewed and confirmed all medical diagnoses by using all available data, including medical record data and assessments from the current study. Diagnoses of HF, stroke, hypertension, atrial fibrillation, transient ischemic attack (TIA), diabetes, myocardial infarction and angina pectoris were set according to ICD-10, while dementia disorders and depressive disorders were diagnosed according to DSM-IV-TR criteria.

### Assessment scales

To assess dependency in personal activities of daily living (ADL), Barthel’s ADL index was used. The scale runs from 0 to 20, with lower scores indicating more dependence in ADL [28].

The Mini Mental State Examination (MMSE), with total scores of 0–30, was used to assess cognition. Higher scores indicate better cognitive function. Scores < 24 indicate cognitive impairment [29].

Nutritional status was assessed using the Mini Nutritional Assessment (MNA). Higher scores on a scale of 0–30 indicate better nutritional status [30].

Depressive symptoms were assessed using the 15-item version of the Geriatric Depression Scale (GDS-15). Total score ranges from 0 to 15, with higher scores indicating more depressive symptoms [31].

The Philadelphia Geriatric Centre Morale Scale (PGCMS) was used to assess the perceived morale dimension of quality of life. On the 17-point scale, higher scores indicate higher morale [32]. After a review by the British Geriatric Society of several quality of life assessment scales, the PGCMS scale was recommended to measure subjective well-being among old people [33].

The first question (“In general, how would you say your health is?”) of the 36-item Short Form Health Survey (SF-36) was used to assess self-rated health. The answers were given on a five-point Likert-type scale: 1 = excellent, 2 = very good, 3 = good, 4 = fair, and 5 = poor [34].

### Statistics

To compare the groups with and without HF, Pearson’s chi-square test was used for dichotomous variables and t-test was used for continuous variables. Descriptive statistics were used to present the prevalence of drug use for T1–T4.

Within the group with HF, the use of selected drug classes at T1–T4 was compared. The World Health Organization Anatomical Therapeutic Chemical Index classification system was used to group the drugs. A multiple logistic regression model was conducted to evaluate time trends in drug use and to control for demographic differences between the samples, with drug class as dependent variable and year of data collection, age, and sex as independent variables. Drug classes included in the analyses were digitalis glycosides (C01A), loop diuretics (C03C), MRAs and other potassium-sparing agents (C03D) (referred to as MRAs throughout this article), BBs (C07), and RAS inhibitors (C09). Multiple logistic regression models were also used to investigate temporal trends in the use of these drug classes within the age groups 85, 90, and ≥95 years. Drug class was the dependent variable, and the year of data collection and sex were included as independent variables for all age groups. For the oldest group (≥95 years), age was also included as an independent variable to adjust for age differences.

To explore if different factors had an impact on drug use over time, another multiple logistic regression model was constructed. In this model, drug class was again the dependent variable, and year of data collection, age, sex, living in a nursing home, MMSE, Barthel’s ADL index, diabetes, hypertension, atrial fibrillation, depressive disorder, stroke and/or TIA, and ischemic heart disease (IHD) were included as independent variables. The diagnoses of stroke and TIA were summarised into one variable, and myocardial heart infarction and angina pectoris were also merged into one variable (IHD). This was done because treatment guidelines for the diagnoses in these groups are very similar.

Version 28 of the SPSS Statistics software package was used for data handling, analysis, and statistical calculations. A p-value of < 0.05 was considered statistically significant.

## Results

The basic characteristics of the study population, assessments, and prevalence of certain diagnoses are presented in Table 1. The three age groups (85, 90, and > 95 years) included 782, 782, and 622 individuals, respectively. Of the total 2186 persons, 654 (29.9%) had HF. The prevalence of HF was 29.6% for women and 30.7% for men, and increased with higher age group (*p* < 0.001). A higher proportion of persons with HF lived in a nursing home (*p* < 0.001) compared to the group who did not have HF. In comparison to those without HF, persons with HF showed lower scores on the assessments of ADL (*p* < 0.001), MNA (*p* < 0.001), and PGCMS (*p* < 0.001), and higher scores on GDS (*p* < 0.001) and SF-36 self-rated health (*p* < 0.001). The diagnoses stroke (*p* = 0.003), atrial fibrillation (*p* < 0.001), diabetes (*p* < 0.001), myocardial infarction (*p* < 0.001), angina pectoris (*p* < 0.001), major neurocognitive disorder (NCD) (dementia according to DSM-IV-TR) (*p* = 0.016), and depressive disorder (*p* < 0.001) were more common in the HF group.

**Table 1:** Basic characteristics, assessments, and diagnoses

	Heart failure *n* = 654 (29.9%)	Without heart failure *n* = 1532 (70.1%)	*p* value
Age category*			< 0.001
Age 85 years, %	21.7 (170/782)	78.3 (612/782)	
Age 90 years, %	30.7 (240/782)	69.3 (542/782)	
Age ≥ 95 years, %	39.2 (244/622)	60.8 (378/622)	
Age, mean ± SD	90.99 ± 4.59	89.58 ± 4.57	< 0.001
Sex*			0.582
Women, %	29.6 (443/1499)	70.4 (1056/1499)	
Men, %	30.7 (211/687)	69.3 (476/687)	
Living in nursing home, %	42.8	29.6	< 0.001
ADL score, mean ± SD	14.81 ± 6.07	16.26 ± 5.88	< 0.001
MMSE score, mean ± SD	20.56 ± 7.32	21.19 ± 7.66	0.117
MNA score, mean ± SD	22.51 ± 4.29	23.38 ± 4.51	< 0.001
GDS score, mean ± SD	3.96 ± 2.66	3.26 ± 2.44	< 0.001
PGCMS score, mean ± SD	11.52 ± 3.08	12.09 ± 3.17	< 0.001
SF-36 question score, mean ± SD	3.39 ± 0.962	3.11 ± 0.951	< 0.001
Stroke, %	26.5	20.0	< 0.001
Hypertension, %	72.9	69.7	0.130
Atrial fibrillation, %	46.0	13.6	< 0.001
TIA, %	13.8	11.3	0.104
Diabetes, %	22.5	12.2	< 0.001
Myocardial infarction, %	25.2	11.4	< 0.001
Angina pectoris, %	43.0	22.5	< 0.001
Major NCD, %	41.3	36.0	0.016
Depressive disorder, %	44.2	35.4	< 0.001

ADL = Activities of Daily Living, GDS = Geriatric Depressions Scale, MMSE = Mini Mental State Examination, MNA = Mini Nutritional Assessment, NCD = Neurocognitive Disorder, PGCMS = Philadelphia Geriatric Centre Morale Scale, SD = Standard Deviation, SF-36 = 36-item Short Form, one question about self-rated health, TIA = Transient Ischemic Attack

^*^For age and sex categories, percentages indicate the proportions of that category with and without heart failure

A description of the heart failure population regarding basic characteristics, assessments and diagnoses for each age group and time point of data collection is presented in Table 2.

**Table 2:** Basic characteristics, assessments and diagnoses for people with heart failure, split by age group at different time points

	2000–2002 (T1)	2005–2007 (T2)	2010–2012 (T3)	2015–2017 (T4)
*Age 85 years*, *n* = *170*				
Women, n (%)	17 (56.7)	17 (56.7)	35 (57.4)	27 (55.1)
Living in nursing home, n (%)	12 (40.0)	10 (33.3)	15 (24.6)	2 (4.1)
ADL score, median (IQR)	20 (16–20)	19 (13–20)	20 (17–20)	20 (19–20)
MMSE score, median (IQR)	26 (18-28.3)	23 (15.5–25.8)	25.5 (21–27)	26 (25–28)
MNA score, median (IQR)	25.5 (20.9–26.6)	24.5 (19.8–25.8)	25.3 (22.5–27.5)	25.3 (22.9–28.0)
GDS score, median (IQR)	4 (3–6)	3 (1-6.5)	3 (2–4)	3 (1–5)
PGCMS score, median (IQR)	11 (9–13)	14 (10–15)	13 (11-14.3)	12 (9.5–14)
SF-36 question, median (IQR)	3.5 (3–4)	3 (3–4)	3 (3–4)	3.5 (3–4)
Stroke, n (%)	7 (23.3)	14 (46.7)	15 (24.6)	11 (22.4)
Hypertension, n (%)	20 (66.7)	22 (73.3)	48 (78.7)	45 (91.8)
Atrial fibrillation, n (%)	12 (40.0)	9 (30.0)	31 (50.8)	24 (49.0)
TIA, n (%)	3 (10.0)	6 (20.0)	9 (14.8)	4 (8.2)
Diabetes, n (%)	8 (26.7)	7 (23.3)	18 (29.5)	14 (28.6)
Myocardial infarction, n (%)	1 (3.3)	8 (26.7)	18 (29.5)	15 (30.6)
Angina pectoris, n (%)	0 (0.0)	17 (56.7)	33 (54.1)	21 (42.9)
Major NCD, n (%)	10 (33.3)	11 (36.7)	21 (34.4)	7 (14.3)
Depressive disorder, n (%)	9 (30.0)	17 (56.7)	19 (31.1)	19 (38.8)
*Age 90 years*, *n* = *240*				
Women, n (%)	32 (76.2)	38 (70.4)	50 (68.5)	42 (59.2)
Living in nursing home, n (%)	22 (52.4)	21 (38.9)	25 (34.2)	15 (21.1)
ADL score, median (IQR)	17.5 (9.8–19)	19 (8.8–20)	19 (15–20)	19 (16.8–20)
MMSE score, median (IQR)	24 (17–26)	22.5 (16.3–25.8)	24 (18–27)	22.5 (18.8–27)
MNA score, median (IQR)	22.75 (19.9–25.1)	23 (19.5–25.5)	25 (23-26.5)	23.5 (18.8–25.5)
GDS score, median (IQR)	4 (2.3-6)	3 (1.5-6)	3 (2–6)	4 (2–5)
PGCMS score, median (IQR)	11 (8–12)	12 (9–14)	12 (10–15)	12 (9–14)
SF-36 question, median (IQR)	4 (4–4)	3 (3–4)	4 (3–4)	3 (3–4)
Stroke, n (%)	14 (33.3)	11 (20.4)	30 (41.1)	21 (29.6)
Hypertension, n (%)	23 (54.8)	40 (74.1)	58 (79.5)	58 (81.7)
Atrial fibrillation, n (%)	17 (40.5)	25 (46.3)	39 (53.4)	40 (56.3)
TIA, n (%)	6 (14.3)	3 (5.6)	10 (13.7)	16 (22.5)
Diabetes, n (%)	8 (19.0)	10 (18.5)	16 (21.9)	18 (25.4)
Myocardial infarction, n (%)	7 (16.7)	16 (29.6)	21 (28.8)	21 (29.6)
Angina pectoris, n (%)	0 (0.0)	26 (48.1)	35 (47.9)	37 (52.1)
Major NCD, n (%)	11 (26.2)	19 (35.2)	25 (34.2)	28 (39.4)
Depressive disorder, n (%)	18 (42.9)	25 (46.3)	36 (49.3)	36 (50.7)
*Age ≥ 95 years*, *n* = *244*				
Women, n (%)	26 (78.8)	42 (85.7)	65 (67.0)	185 (75.8)
Living in nursing home, n (%)	25 (75.8)	35 (71.4)	62 (63.9)	36 (55.4)
ADL score, median (IQR)	17 (4.3–17)	15 (6.8–18)	16 (8-17.5)	18 (9.3–19)
MMSE score, median (IQR)	23 (8.3–23.8)	18 (13–23)	19 (13.5–23.5)	22 (15.5–25)
MNA score, median (IQR)	25 (15.3–25.4)	22 (19-24.9)	24 (21–25)	22 (18–24)
GDS score, median (IQR)	4 (2-6.3)	5 (2-5.5)	4 (2–5)	4 (2-5.75)
PGCMS score, median (IQR)	11 (6.8–12.5)	11 (8–13)	12 (11-13.5)	11 (8.3–14)
SF-36 question, median (IQR)	4 (2.5-4)	3 (3–4)	3 (3–4)	4 (3–4)
Stroke, n (%)	5 (15.2)	11 (22.4)	21 (21.6)	13 (20.0)
Hypertension, n (%)	10 (30.3)	28 (57.1)	70 (72.2)	55 (84.6)
Atrial fibrillation, n (%)	16 (48.5)	15 (30.6)	37 (38.1)	36 (55.4)
TIA, n (%)	3 (9.1)	7 (14.3)	16 (16.5)	7 (10.8)
Diabetes, n (%)	8 (24.2)	8 (16.3)	15 (15.5)	17 (26.2)
Myocardial infarction, n (%)	0 (0.0)	16 (32.7)	26 (26.8)	16 (24.6)
Angina pectoris, n (%)	0 (0.0)	26 (53.1)	48 (49.5)	38 (58.5)
Major NCD, n (%)	17 (51.5)	27 (55.1)	61 (62.9)	33 (50.8)
Depressive disorder, n (%)	11 (33.3)	21 (42.9)	44 (45.4)	34 (52.3)

ADL = Activities of Daily Living, GDS = Geriatric Depressions Scale, IQR = Interquartile Range, MMSE = Mini Mental State Examination, MNA = Mini Nutritional Assessment, NCD = Neurocognitive Disorder, PGCMS = Philadelphia Geriatric Centre Morale Scale, SD = Standard Deviation, SF-36 = 36-item Short Form, one question about self-rated health, TIA = Transient Ischemic Attack

### Differences in drug treatment over time among persons with HF

**Fig. 1:**
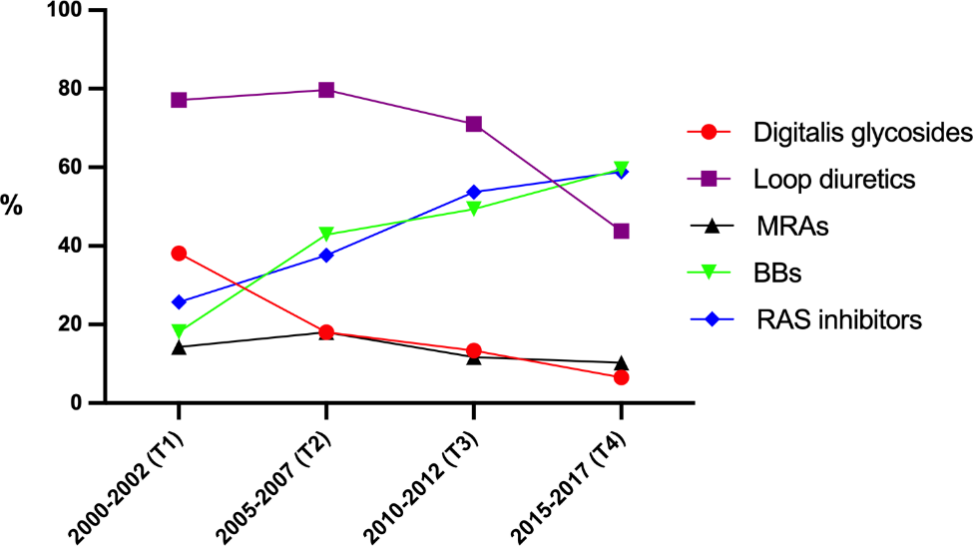
Drug use over time among all people with heart failure BBs = beta-blockers, MRAs = mineralocorticoid receptor antagonists, RAS = renin-angiotensin system

Use of the different drug classes in the HF group at T1–T4 is presented in Table 3 and illustrated in Fig. 1. Corrected for age and sex, the use of RAS inhibitors and BBs increased over time (*p* < 0.001), whereas the use of digitalis and loop diuretics decreased (*p* < 0.001). At T2, the use of loop diuretics and MRAs was higher than at T1, but the overall trends were declining. However, the trend for MRAs was not statistically significant. The logistic regression model including potential confounders is presented in Table 4.

**Table 3:** Drug use at T1 (2000–2002), T2 (2005–2007), T3 (2010–2012), and T4 (2015–2017), including odds ratios (ORs) with 95% confidence intervals (CIs) for the trend over time

	2000–2002 (T1)	2005–2007 (T2)	2010–2012 (T3)	2015–2017 (T4)	OR per year (95% CI)*	*p* value for trend*
People with heart failure, n (%)	105 (24.5)	133 (28.4)	231 (35.6)	185 (29.0)		
Digitalis glycosides, n (%)	40 (38.1)	24 (18.0)	31 (13.4)	12 (6.5)	0.864 (0.828–0.901)	< 0.001
Loop diuretics, n (%)	81 (77.1)	106 (79.7)	164 (71.0)	81 (43.8)	0.899 (0.868–0.931)	< 0.001
MRAs, n (%)	15 (14.3)	24 (18.0)	27 (11.7)	19 (10.3)	0.963 (0.922–1.006)	0.087
BBs, n (%)	19 (18.1)	57 (42.9)	114 (49.4)	110 (59.5)	1.123 (1.086–1.161)	< 0.001
RAS inhibitors, n (%)	27 (25.7)	50 (37.6)	124 (53.7)	109 (58.9)	1.107 (1.072–1.144)	< 0.001

*Corrected for age and sex

BBs = beta-blockers, MRAs = mineralocorticoid receptor antagonists, RAS = renin-angiotensin system

**Figs. 2:**
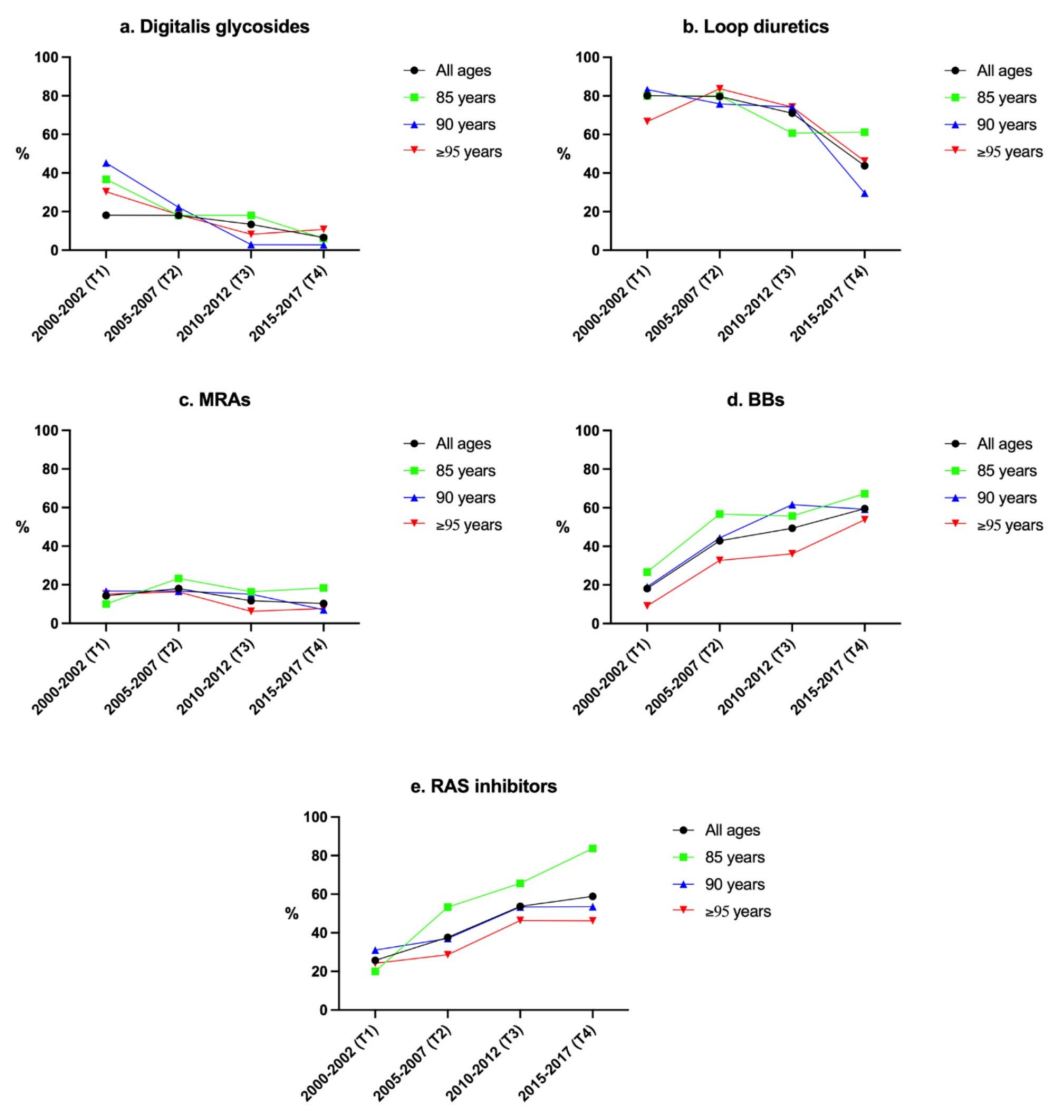
**a–e**. Temporal trends in drug use over time in different age groups among very old people with heart failure (not statistically tested) BBs = beta-blockers, MRAs = mineralocorticoid receptor antagonists, RAS = renin-angiotensin system

### Drug treatment over time in different age groups

Figure 2a–e and sTables 1a–c in the online supplement present the prevalence of drug classes for the three age groups 85, 90, and > 95 years. The oldest group appeared to have lower treatment rates of MRAs, BBs, and RAS inhibitors.

### Factors associated with the use of drug classes

In the logistic regression model including potential confounders (age, sex, living in a nursing home, ADL, MMSE, diabetes, hypertension, atrial fibrillation, depressive disorder, stroke and/or TIA, and IHD), the time trends in use of digitalis, loop diuretics, BBs, and RAS inhibitors persisted (Table 4 and online supplement sTable 2).

Higher age was associated with lower use of RAS inhibitors and BBs. Women were more likely to be prescribed RAS inhibitors than men, and higher ADL was associated with increased BB use. Persons with atrial fibrillation were more likely to use digitalis and RAS inhibitors. People with hypertension had a higher use of BBs and RAS inhibitors. Diabetes was associated with a higher prevalence of loop diuretics and RAS inhibitors. IHD was associated with higher prescribing of BBs but lower use of digitalis. Persons with depressive disorder were less likely to receive MRA treatment.

**Table 4:** Multiple logistic regression analysis of factors associated with drug classes among people with heart failure. Data are only shown for statistically significant results. The complete analysis is available in sTable 2 in the online supplement

	OR	95% CI	*p* value
**Digitalis glycosides**			
Year of data collection	0.866	0.815–0.921	< 0.001
Atrial fibrillation	7.533	4.018–14.123	< 0.001
IHD*	0.508	0.277–0.932	0.029
**Loop diuretics**			
Year of data collection	0.945	0.901–0.990	0.018
Diabetes	2.161	1.206–3.870	0.010
**MRAs**			
Depressive disorder	0.494	0.271–0.899	0.021
**BBs**			
Age	0.938	0.892–0.986	0.011
Year of data collection	1.074	1.028–1.123	0.001
ADL	1.063	1.003–1.127	0.041
Hypertension	1.830	1.125–2.978	0.015
IHD*	2.300	1.508–3.510	< 0.001
**RAS inhibitors**			
Age	0.941	0.895–0.989	0.016
Female sex	1.684	1.077–2.632	0.022
Year of data collection	1.110	1.061–1.160	< 0.001
Diabetes	1.903	1.161–3.121	0.011
Hypertension	1.732	1.066–2.814	0.026
Atrial fibrillation	1.892	1.248–2.868	0.003

ADL = Activities of daily living, BBs = beta-blockers, CI = confidence interval, IHD = ischemic heart disease, MRAs = mineralocorticoid receptor antagonists, OR = odds ratio, RAS = renin-angiotensin system

*IHD includes the diagnoses myocardial infarction and angina pectoris

## Discussion

In this analysis of cross-sectional data at four different time points between 2000 and 2017 among very old people, the major findings were that the use of the HF drugs RAS inhibitors and BBs increased over time, while the use of loop diuretics and digitalis decreased. In this population, the prevalence of HF was about 30%, irrespective of sex, and the prevalence of HF increased with age.

### Description of the very old HF population

In common with other studies focusing on people ≥80 years old with HF, a majority of our participants were female [13, 24, 35]. Our finding of higher prevalences of cardiovascular comorbidities and diabetes among persons with HF is consistent with previously published literature [2, 36, 37]. Similar results are seen in the literature regarding higher prevalence of major NCD [36–40] and depressive disorder among people with HF [36, 40–42]. Malnutrition is also common among people with HF [43, 44], and in our material, the participants with HF had poorer nutrition status. In addition, quality of life has been reported to be lower among people with HF than the general population [1, 45, 46], which aligns with worse outcomes on the PGCMS scale and the SF-36 question about self-rated health in our HF population.

### Temporal trends in HF treatment among the very old

A few previous studies have examined trends in HF drug treatment over time with a focus on, or subgroup analyses of, people ≥80 years old [13, 21, 24, 47]. The only conclusive change seen in all articles was increased prescribing over time for BBs, as in our results. Moreover, the use of digitalis decreased over time both in our results and in the studies where this drug class was included [24, 47]. This is in line with a long-term trend that has been seen in patients regardless of age [47–51].

For treatments other than BBs and digitalis, the results in the abovementioned articles were diverse. Parén et al. used the Swedish National Patient Register (NPR) and the Swedish Prescribed Drug Register to analyse HF drug use during the period 2005–2014 [47]. The NPR has a 99% coverage of all hospitalisations in Sweden [52]. A subgroup analysis was performed for the 27 662 participants who were 85–99 years old. During this time interval, the use of RAS inhibitors increased while the use of MRAs and loop diuretics decreased. Another study showed that the use of RAS inhibitors decreased and MRAs showed no statistically significant change over time for people > 80 years old [21]. That study selected all persons with EF < 40% from the Swedish HF Registry as their study population, and compared HF drug use over the years 2000–2018 among the age groups > 80, 70–79, and < 70 years. One study that recruited hospitals from 30 ESC countries showed that the use of both RAS inhibitors and MRAs was higher in 2005 than in 2000 [24]. That study compared HF treatment for people > 80 years old between the years 2000 (Euro Heart Failure Survey I, EHFS I) and 2005 (Euro Heart Failure Survey II, EHFS II).

One important factor that might explain the differences in the results is the time span studied. The material in Stolfo et al. and Parén et al. [21, 47] covered roughly the same years as our material, while Komajda et al. [24] looked at the early 2000s. The landmark study for MRAs, RALES, was published in 1999 [53]. Sharp increases were seen in the prescribing rates of MRAs in the years following the publication of RALES, but problems emerged with adverse events such as hyperkalaemia [54]. The benefits compared to the risks of using MRAs in the community were questioned [55, 56], which could explain the subsequent decline in prescribing of MRAs. This could also be the reason for the peaks in MRA prevalence found to occur in 2004 by Stolfo et al. [21], in 2005 by Komajda et al. [24], in 2006 by Parén et al. [47], and in 2005–2007 in our results; these peaks were followed by decreasing levels in all studies (except Komajda et al., whose study ended in 2005). The time factor could also explain divergent findings regarding whether or not the use of loop diuretics decreased. As guidelines have been implemented to a greater extent over time, resulting in higher use of evidence-based drug classes, the need for loop diuretics may not be as pronounced as before.

The differences in study populations could also affect the comparability of the studies.

All participants in Stolfo et al. had EF < 40% [21], while we and other studies included participants irrespective of EF [13, 24, 47]. Since there were no evidence-based treatment recommendations for HF with preserved EF (HFpEF) during the time span studied [19], this could be a contributing factor to the relatively low levels of RAS inhibitors and BBs among our participants. The higher prevalence of HFpEF among old people with HF has been suggested as an explanation for the discrepancies regarding the use of evidence-based treatment between younger and older persons [8]. In a population comprising all patients with a diagnosis of HF between 2010 and 2019 from the Heart Centre or Department of Internal Medicine at Umeå University Hospital in northern Sweden, 47% had HFpEF [57]. In our very old population, the prevalence of HFpEF was possibly even higher. As a consequence of not knowing the proportions with different classes of EF, it is not possible to state which levels of evidence-based medications are satisfactory. Nevertheless, recently published studies have shown that old people with HF are under-prescribed evidence-based HF treatment even in populations limited to HFrEF [7, 21, 58]. Moreover, even after adjusting for additional factors such as comorbidities, NYHA classification, and renal function, the age differences in receiving guideline-recommended treatment were still present in these studies [21, 58]. In our material, the use of evidence-based HF medications appeared to be lowest in the oldest group (≥95 years). However, the differences between age groups were not statistically tested.

The importance of person-centred therapy must also be underlined, and taken into consideration when interpreting the results, as very old people with HF have a high prevalence of comorbidities, polypharmacy, frailty, and risk of adverse medication events, which may affect the possibility of following guidelines [6]. Extra attention regarding drug treatment is required among people ≥80 years with HF, due to these factors as well as declining renal function with age, and so dose reductions may be needed [59]. We can speculate that the prescribers prioritised RAS inhibitors among people with very low renal function who may not have tolerated treatment with both RAS inhibitors and MRAs, resulting in very low levels of MRAs among the oldest in our population. It might have been a challenge to start treatment with MRAs in this group due to the risk of adverse events like hyperkalaemia.

The overall increasing levels of evidence-based HF treatment among very old people over time, seen in our material and previously published studies, may be an indication of increased awareness among prescribers of the age differences and an attempt to even these out.

### Factors associated with drug classes

Atrial fibrillation was strongly associated with digitalis in our material. This is logical, as this drug class has long been recommended as a rate-controlling substance for the condition [60]. Unexpectedly, atrial fibrillation was not associated with BBs; however, as expected, IHD was associated with a higher prevalence of BBs. This reflects the treatment guidelines, where BBs have been recommended to most patients with stable angina or after a myocardial infarction [61, 62]. Associations were also found between hypertension and both BBs and RAS inhibitors, again reflecting the guidelines where people with hypertension and HF have been recommended to use RAS inhibitors and BBs [63]. RAS inhibitors were suggested as the first-line treatment of hypertension among persons with HF and/or diabetes as early as the beginning of the 2000s [64], which aligns with the association found between diabetes and RAS inhibitors. Diabetes was also associated with higher use of loop diuretics. In a previous Danish study, a suggested explanation was that increased use of loop diuretics, which can be seen as a reflection of the severity of HF, was a risk factor for developing diabetes [65]. However, since we do not know if the use of loop diuretics preceded the diagnosis of diabetes, we were not able to determine whether there was a cause-effect association in our population.

Age was associated with lower use of BBs and RAS inhibitors. Under-prescribing of BBs and RAS inhibitors to old people has been reported in several articles. Patient-related factors such as comorbidities, poor tolerance, or frailty, as well as prescriber-related factors such as lack of awareness of guidelines, fear of adverse effects, reluctance to make changes in very old people’s treatment, and focus on improved symptoms rather than outcome, have been suggested to explain why these drugs are less used among old people [7, 9, 24, 58, 66–68].

There was an association between female sex and higher prescribing of RAS inhibitors in our material. Similar results have been shown in a recent Swedish study [21], the authors of which hypothesised that sex-related differences in patient characteristics could explain the discrepancy. Older age, higher heart rate, higher blood pressure, and more symptoms have been shown among women compared to men in the Swedish HF registry. Aside from older age, which is not relevant in the present study as we adjusted for age, this might be the case in our material as well.

An association between depressive disorders and lower use of MRAs was seen in our results. While one might speculate that individuals with depression could be less interested in proceeding to more intense treatment for their other disorders, this finding could also represent a chance association.

### Strengths and limitations

As there were no exclusion criteria connected to health status, cognitive function, or living conditions in the GERDA study, our population is expected to be representative of the sampled age groups in northern Sweden. The population register from the Swedish National Tax Agency is highly accurate, which made it easy to identify and contact eligible participants. The sample size is large in the context of the very old age group, and a relatively high proportion of the invited persons chose to take part. However, we selected people aged 85, 90, and ≥95, and cannot say for sure that these groups reflect the heterogeneity of all individuals aged ≥85.

Trained investigators thoroughly conducted all tests and assessments of the participants. To prevent loss of data, information regarding health and medication use was collected directly from the participants or someone very close to them, in addition to the information that was extracted from medical records.

We acknowledge that there are limitations in our study, the major one being the lack of information regarding EF and NYHA classes. These factors are the most important to determine which HF treatment is recommended. Therefore, we cannot tell how many of the participants should have been treated with the drugs that are recommended for HFrEF. Also, since this data collection was initiated already in the year 2000, the conditions and opportunities for diagnosing HF may have changed over time, which in turn could affect the prevalence of HF.

Other important factors where information was not accessible are impaired renal function and risk of hypotension or bradychardia. This could limit the use of HF medications in our population, and must be considered when interpreting the results.

Information regarding use of *pro re nata* medication was not available. Among the drug classes investigated, loop diuretics can be recommended as such, and so there is a risk of under-reporting of loop diuretics in our material. Regarding the trend over time, if the decline in the use of loop diuretics was partly based on an increased *pro re nata* use later in time, this would still be a sign of improved treatment, as loop diuretics should be used in as low doses as possible, and self-management is encouraged to treat fluid retention according to guidelines [1, 14–19]. Further, the use of other diuretics might have had an impact in the use of loop diuretics, which should be taken into account when reading the results.

It was not possible to obtain information regarding how many participants used MRAs from the group with potassium-sparing agents. We assume that MRAs accounted for most of the drug use in the group, but this could be an overestimation of MRA users.

## Conclusion

In our material, comprising very old people, about 30% of both men and women had HF. The prevalence of HF increased with higher age. Both cardiovascular and non-cardiovascular comorbidities were more common among the persons with HF compared to those without HF, and those with HF also reported lower quality of life.

In the HF population, the use of evidence-based medications for HF increased over time. Between 2000 and 2017, there was an increase in the use of RAS inhibitors and BBs among persons with HF, in parallel with a decrease in the use of loop diuretics and digitalis. The improved use of HF drugs over time may be a sign of better awareness among prescribers regarding the under-prescribing of guidelines-recommended treatment to old people.

Among several factors associated with cardiovascular drug use in the HF population, we found that age correlated with a lower prevalence of RAS inhibitors and BBs. This may be an indication that under-prescribing to some extent remains. However, the lower use of these medications could also indicate cautious prescribing to very old and frail individuals, and while it might be possible to further improve prescribing it is also increasingly important to individually tailor drug treatment in advanced age.

### Electronic supplementary material

Below is the link to the electronic supplementary material.

Supplementary Material 1

## Data Availability

The datasets generated and/or analysed during the current study are not publicly available due to issues of individual privacy but are available from the corresponding author on reasonable request.
